# Patterns of Ultra-Processed Food Consumption in a Gluten-Free Diet: A Target for Nutritional Intervention

**DOI:** 10.3390/nu18132173

**Published:** 2026-07-04

**Authors:** Teresa Nestares, María Jiménez-Muñoz, Marta Flor-Alemany, Marta Herrador-López, Lara Bossini-Castillo, Irene Zapata-Martínez, Víctor Manuel Navas-López, Rafael Martín-Masot

**Affiliations:** 1 Departamento de Fisiología, Facultad de Farmacia, Universidad de Granada, 18071 Granada, Spain; nestares@ugr.es; 2 Instituto de Nutrición y Tecnología de los Alimentos “José Mataix Verdú” (INYTA), Centro de Investigación Biomédica (CIBM), Universidad de Granada, 18071 Granada, Spain; rafammgr@gmail.com; 3 Pediatric Unit, Hospital la Serranía, 29400 Ronda, Spain; mariajmnzm@gmail.com; 4 Department of Nutrition and Food Science, Campus of Melilla, University of Granada, 52001 Melilla, Spain; floralemany@ugr.es; 5 Sección de Gastroenterología y Nutrición Infantil, Hospital Regional Universitario de Málaga, 29010 Málaga, Spain; herradorlopezm@gmail.com (M.H.-L.); victor.navas@gmail.com (V.M.N.-L.); 6 Departamento de Genética, Instituto de Biotecnología, Centro de Investigación Biomédica (CIBM), Universidad de Granada, 18071 Granada, Spain; 7 Reproducción Humana y Enfermedades Hereditarias y Complejas (IBS-TEC14), Terapias Avanzadas y Tecnologías Biomédicas, Instituto de Investigación Biosanitaria de Granada (ibs.GRANADA), 18012 Granada, Spain; 8 Departamento de Farmacología y Pediatría, Facultad de Medicina, Universidad de Málaga, 29071 Málaga, Spain; irenezapata365@uma.es

**Keywords:** celiac disease, children, ultraprocessed foods, gluten-free diet

## Abstract

**Background/Objectives**: Celiac disease (CD) is a complex multifactorial disorder driven by genetic susceptibility and environmental triggers, with ultra-processed foods (UPFs) acting as potential disruptors of immune homeostasis. This study aimed to characterize the patterns and temporality of UPF consumption in a pediatric population with CD to provide evidence-based insights that can optimize the nutritional quality of a gluten-free diet (GFD) beyond mere gluten avoidance. **Methods**: A total of 128 children aged 5–14 years were enrolled, comprising a baseline cohort of 48 children newly diagnosed with CD (pre-GFD), 88 patients who had followed a GFD for at least 6 months (post-GFD), including 44 participants from the pre-GFD cohort prospectively re-evaluated after 12 months and 44 additional patients with established GFD adherence and a control group of 36 healthy children (CTRL). Dietary intake was assessed using three-day 24 h recalls. Food processing levels were determined using the NOVA classification system, and adherence to the Mediterranean Diet was evaluated via the KIDMED index. **Results**: At baseline, UPFs (NOVA 4) were the primary daily energy source for both celiac patients and controls, accounting for over 57% of total caloric intake, peaking during breakfast (~74%) and afternoon snacks (~81%). Longitudinal analysis showed that the nutritional profile and global UPF consumption remained remarkably stable after 12 months on a GFD, though a significant increase in vitamin B6 intake was observed (0.9 ± 0.4 vs. 1.1 ± 0.5 mg; *p* = 0.034). However, meal-pattern shifts occurred over the 12 months: celiac children significantly reduced their daily intake of culinary ingredients (NOVA 2; *p* = 0.029) and processed foods (NOVA 3; *p* = 0.025). Compared to healthy controls, post-GFD patients exhibited significantly lower Vitamin D intakes (4.6 ± 9.4 vs. 6.2 ± 12.3 µg/day; *p* = 0.008), meeting only 30.8% of the reference intake. Both groups presented inadequate intakes of iron, calcium, folate, magnesium, and zinc. **Conclusions**: Pediatric celiac patients exhibit a high, deeply ingrained consumption of UPFs that mirrors healthy controls and persists 12 months after starting a GFD. While the GFD alters meal processing dynamics, it fails to resolve baseline micronutrient insufficiencies and is associated with lower dietary vitamin D intake, highlighting the urgent need for targeted nutritional interventions that focus on whole food quality rather than just gluten elimination.

## 1. Introduction

Celiac disease (CD) is included in the group of complex or multifactorial diseases, i.e., those caused by the interaction of genetic and environmental factors [1]. Thus, subjects genetically susceptible to CD can experience a loss of oral tolerance to gluten at any time in their life without the cause currently being known [2]. Nevertheless, there are factors in the spotlight, such as intestinal dysbiosis, which are known to induce the alteration of tight junctions in the epithelium and increase permeability, allowing gluten peptides to enter the lamina propria, and facilitating an immune response [3].

Interestingly, the interaction between feeding practices and subsequent induced changes in the composition of the gut microbiota appear to play a role in the development of CD [3]. Thus, in a situation of intestinal homeostasis, there is tolerance or an absence of immune response to dietary proteins [4]. However, environmental factors that alter this microbiota and, therefore, intestinal homeostasis, can affect immune function, leading to proinflammatory reactions, to antigens that would otherwise be innocuous, and to the development of chronic inflammation [5]. In this regard, current diets based on processed and ultra-processed foods (UPFs) have been hypothesized to contribute to intestinal dysbiosis, promoting a proinflammatory response, an increase in intestinal permeability, and susceptibility to autoimmune diseases, such as CD, in individuals with a genetic predisposition [6].

The fundamental pillar of CD treatment is strict compliance with a gluten-free diet (GFD), whose nutritional adequacy in celiac children who follow it is a matter of controversy [7,8]. The need for the GFD to be, in addition to gluten-free, nutritionally adequate is already well established, although there is a scarcity of evidence-based guidelines on the dietary management of CD through the GFD beyond safety [8]. Moreover, our recent studies are revealing new aspects of the role of this diet, when adequate, on the evolution of the disease, such as its immunomodulatory and anti-inflammatory effect [9,10]. Therefore, in children with CD and even in those with genetic susceptibility to CD, dietary monitoring and control are especially important to avoid those components of the GFD that may promote the immune and inflammatory response related to the disease, as is the case of UPFs [11]. To this end, and once we are aware of their high consumption [12,13], we consider it of great interest to know what the dietary habits related to the consumption of UPFs are in order to combat them.

Childhood is a fundamental stage for health in adulthood. Moreover, dietary habits acquired in childhood and adolescence prevail in adulthood. Therefore, a more detailed characterization of modifiable factors, such as diet quality, is of clinical and public health interest. Therefore, the objective of this work has been to study the temporality of UPF consumption in a pediatric population with CD to understand a pattern that allows us to implement an effective strategy to improve the diet quality of this population group and not limit ourselves to gluten avoidance.

## 2. Materials and Methods

### 2.1. Study Design and Participants

A total of 128 children aged 5–14 years were enrolled in the study. Participants were recruited between March 2023 and September 2025, and CD diagnosis was made based on the criteria from the European Society of Pediatric Gastroenterology, Hepatology, and Nutrition (ESPGHAN) [14].

The study sample comprised three distinct clinical groups: (1) a cohort of 48 children newly diagnosed with CD evaluated at baseline before initiating a GFD (pre-GFD), (2) 88 children with CD who had followed a GFD for at least 6 months (post-GFD), including 44 participants from the newly diagnosed cohort who were reassessed after 12 months and 44 additional patients with established GFD adherence (3) a control group consisting of 36 healthy children (CTRL). These children presented with minor symptoms related to chronic functional constipation, according to the Rome IV criteria [15], or other non-gastrointestinal issues.

For the established GFD group, a minimum diet duration of six months was required to ensure that participants had completed the initial dietary adaptation period and had established relatively stable food-selection and family dietary routines. The inclusion criteria for the control group were age between 5 and 14 years, absence of serum IgA anti-transglutaminase (tTG) antibodies, normal weight for their age, no gastrointestinal disorders in the previous year and a normal appetite. Exclusion criteria for both groups included liver or kidney diseases, acute or chronic inflammation, inflammatory bowel disease, diabetes, chronic asthma and the use of dietary supplements with antioxidant properties. Additionally, patients with obesity (as defined by the International Task Force criteria) [15,16] and those refusing to sign the informed consent were excluded.

Written informed consent was obtained from all parents. The study was conducted according to the guidelines of the Declaration of Helsinki, and approved by Comité de Etica de la Investigación (CEIC) Provincial de Málaga (Ref. 1875-N-22) on 30 May 2023.

### 2.2. Clinical and Socio-Demographics Characteristics

Participants’ clinical and socio-demographic characteristics (i.e., age, household composition, parents’ marital status, educational level and smoking habit) were assessed by the same group of researchers.

### 2.3. Anthropometric Measures

Anthropometric characteristics (weight, height) were assessed in the control and the celiac subjects. Height was measured to the nearest 5 mm using a stadiometer (Seca 22, Hamburg, Germany). Body weight was measured using the same mechanical balance (Seca200, Hamburg, Germany). Height and weight were used to calculate the BMI (weight [kg]/height [m]^2^).

### 2.4. Dietary Assessment

Dietary intake was assessed using a 24 h recall over three days, including one non-working day. Parents or guardians recorded the food and beverages consumed by the children during the different meals (breakfast, morning snack, lunch, afternoon snack, dinner), noting quantities and brands. Household measurements were converted to grams using an equivalence table. The data were analyzed using the Evalfinut 2.0 software, which includes the Spanish food composition database (BEDCA, accessed on 30 September 2025) [17] and the United States Department of Agriculture database (FoodData Central, accessed on 30 September 2025) [18]. Both were supplemented with nutritional information from gluten-free products. The program calculated energy intake, the proportion of macro and micronutrients, and the adequacy of intake according to age, physical activity and BMI recommendations.

Then we proceeded to classify foods according to the NOVA classification, the most frequently used method to examine diets according to food processing, which has been employed in many studies conducted in several countries [19]. It is also recognized and used by international bodies including PAHO, WHO and FAO [20,21]. NOVA is a food classification based on the extent and purpose of industrial food processing, which divides foods into four groups: unprocessed and minimally processed foods, processed culinary ingredients, processed foods and ultra-processed foods [22]. This last category comprises a group of industrial formulations that are manufactured using several ingredients and a series of processes. The three-day food records were analyzed in order to classify the foods as unprocessed or minimally processed, culinary ingredients, processed and ultraprocessed foods based on the NOVA classification [22]. The total dietary energy intake was calculated for each individual. Subsequently, the energy (kcal/day) and percentage of calories (% of the total daily energy intake) derived from each category of the NOVA classification food item was calculated. All diaries were analyzed by the same trained nutritionist using the data were analyzed using the Evalfinut version 2.0 software, which includes the BEDCA [17]. The specific composition of gluten and gluten-free UPF (from labels) were introduced in the software when calculating dietary balance. The mean of the three-day food records was employed in the present analyses.

Moreover, the Mediterranean Diet Quality Index in Children and Adolescents (KIDMED) survey was employed to assess adherence to the Mediterranean diet (MD) [23]. This index is determined by a 16-point questionnaire that assesses various dietary habits. Each answer is scored according to whether it is consistent with habits associated with the Mediterranean dietary pattern, and scores are added up to quantify the total index of the subject’s adherence to the MD. The KIDMED index ranges from 4 (no adherence to the MD) to 12 (complete adherence to the MD).

### 2.5. Data Analyses

The baseline characteristics of the study sample were described using descriptive statistics (mean ± standard deviation) for quantitative variables and the percentage of participants (*n*, %) for categorical variables. The Chi-square test and Wilcoxon rank-sum test were used to explore differences in categorical and continuous baseline variables, respectively.

A one-way analysis of covariance (ANCOVA) after adjustment for age and sex was employed to assess differences in total energy intake between celiac patients and healthy controls. After controlling for age and sex, and with additional adjustment for total energy intake (kcal/day), ANCOVA was used to assess macro- and micronutrient intakes, reference intake percentages (% RI) and daily or meal-specific energy contributions from NOVA food processing categories between groups. The same adjusted ANCOVA approach was utilized to evaluate correlations between socioeconomic monthly income and NOVA categories. For longitudinal analysis within the celiac cohort (*n* = 41), Linear Mixed-Effects Models (LMM) adjusted for age, sex, and total energy intake were applied to compare dietary evolution from baseline to the 12-month follow-up.

R software (version 4.5.2) was used to analyze the data, and the statistical significance was set at *p* < 0.05.

## 3. Results

### 3.1. Baseline Sociodemographic and Clinical Characteristics

The experimental groups consisted of 36 healthy controls and 88 patients with CD. No significant differences were observed regarding sex distribution (*p* > 0.9), number of siblings (*p* = 0.6), or monthly income (*p* > 0.9). However, significant disparities were found in residential distribution, with the control group being predominantly rural (69% vs. 33%; *p* = 0.017). Regarding anthropometric markers, the CD group was significantly younger (9.0 ± 3.2 vs. 12.4 ± 2.5 years; *p* = 0.029) and exhibited a higher BMI (18.5 ± 2.8 vs. 17.1 ± 3.1 kg/m^2^; *p* = 0.025) compared to controls. No significant differences were found for height or weight (Table 1).

### 3.2. Baseline Nutritional Intake and Mediterranean Diet Adherence

Nutritional analysis showed no significant differences in total energy intake (1806.7 ± 351.2 vs. 1850.7 ± 460.0 kcal/day; *p_adj_* = 0.256) or macronutrient distribution (protein, total fat, and carbohydrates) between groups. Although unadjusted analyses initially suggested lower iron (*p* = 0.034) and sodium (*p* = 0.046) intake in the Celiac group, these differences were no longer significant after adjusting for age, sex, and total energy intake (*p_adj_* > 0.1) (Table S1). Similarly, no significant disparities were found in the intake of vitamins (A, B-group, C, D, and E) or other minerals after adjustment. Both groups showed comparable levels of adherence to the Mediterranean Diet, with mean scores of 6.3 ± 2.1 for controls and 6.4 ± 2.2 for celiac patients (*p_adj_* = 0.078).

### 3.3. Baseline Food Processing Levels (NOVA)

Ultra-processed foods (NOVA 4) were the predominant energy source in both groups, accounting for 57.3% of daily energy in celiac patients (Table 2). While daily distribution was similar, meal-specific differences were observed; celiac patients consumed significantly less energy from processed foods (NOVA 3) during breakfast compared to controls (3.2% vs. 11.4%; *p_adj_* = 0.007). Notably, the energy contribution of UPFs was highest during the afternoon snack (81.9%) and breakfast (74.1%) for the celiac group (Table 2).

### 3.4. Longitudinal Evolution of Nutritional Intake (12-Month GFD)

**Figure 1:**
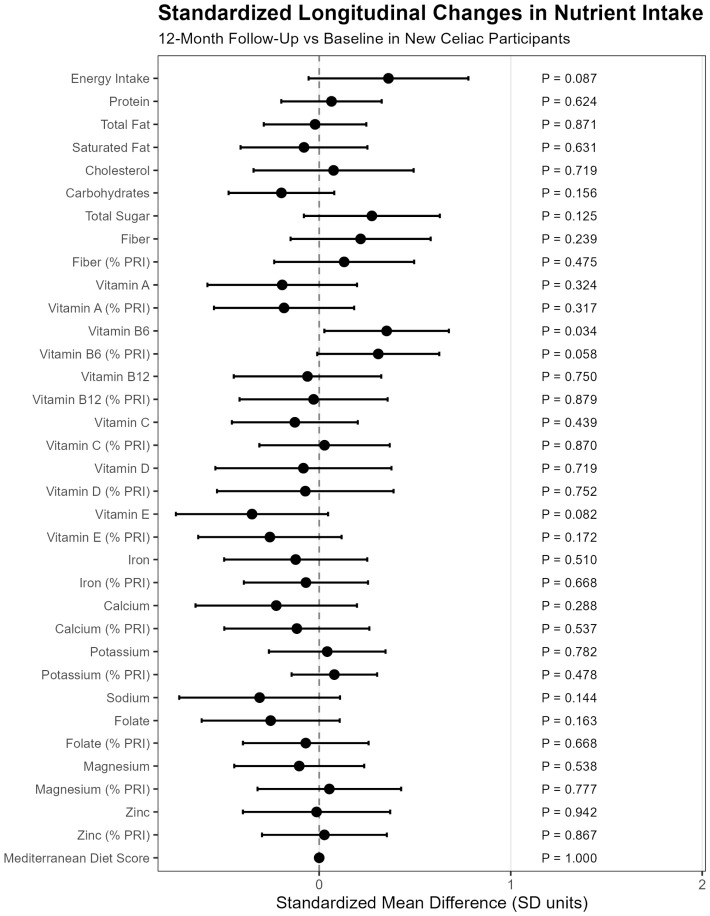
Standardized longitudinal changes in nutrient intake and Mediterranean Diet Score (12-month follow-up vs. baseline). Data are presented as Standardized Mean Differences (SMD) in units of standard deviation with 95% confidence intervals (CI). *p*-values were calculated using Linear Mixed-Effects Models (LMM) adjusted for sex, age, and (for nutrients) total energy intake. This standardization allows for the direct visual comparison of nutrients measured in different units. PRI: Population Reference Intake based on EFSA guidelines.

Longitudinal analysis of celiac patients (*n* = 41) revealed a remarkably stable nutritional profile after 12 months on a gluten-free diet (Figure 1). No significant changes were observed in total energy intake, macronutrient distribution, or Mediterranean Diet adherence (6.2 at both time points). Regarding micronutrients, a significant increase in Vitamin B6 intake was identified (0.9 ± 0.4 vs. 1.1 ± 0.5; *p_adj_* = 0.034). Although unadjusted data suggested an increase in total sugar consumption (*p* = 0.044), this trend did not reach statistical significance after adjusting for age, sex, and total energy intake (*p_adj_* = 0.125). No other significant disparities were found in mineral or vitamin adequacy during the follow-up period (Figure 1).

### 3.5. Evolution of Meal Patterns and Food Processing

After 12 months on a GFD, celiac patients significantly reduced their daily intake of culinary ingredients (NOVA 2: 9.2 vs. 6.4%; *p_adj_* = 0.029) and processed foods (NOVA 3: 13.3% vs. 9.8%; *p_adj_* = 0.025). While daily consumption of ultra-processed foods (NOVA 4) showed an upward trend, it did not reach statistical significance (54.6% to 58.9%; *p_adj_* = 0.173).

**Figure 2:**
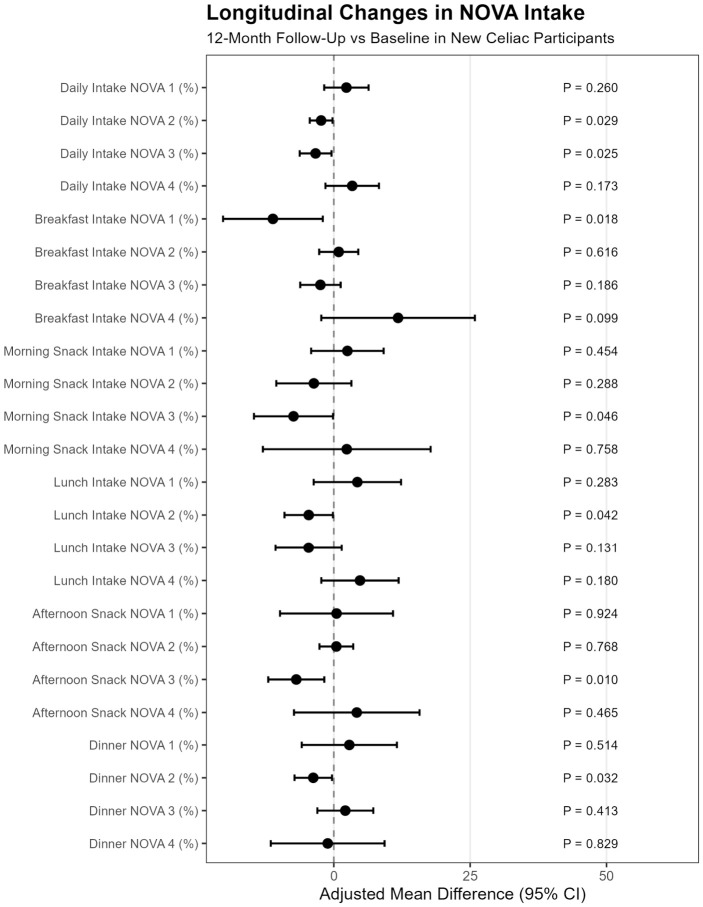
Longitudinal Changes in NOVA Food Classification and Daily Distribution in Celiac Patients. Data are expressed as adjusted mean differences with 95% confidence intervals (CI). *p*-values were calculated using Linear Mixed-Effects Models (LMM) adjusted for sex, age, and total energy intake. NOVA 1: Unprocessed or minimally processed foods; NOVA 2: Processed culinary ingredients; NOVA 3: Processed foods; NOVA 4: Ultra-processed foods.

At breakfast, consumption of unprocessed foods (NOVA 1) decreased markedly (21.1% to 9.0%; *p_adj_* = 0.018). In contrast, a significant reduction in processed foods (NOVA 3) was observed during mid-morning snacks (*p_adj_* = 0.046) and afternoon snacks (*p_adj_* = 0.010). Additionally, the use of culinary ingredients (NOVA 2) during lunch and dinner decreased significantly (*p_adj_* < 0.05) over the follow-up period (Figure 2).

### 3.6. Comparison Between Patients with Established GFD and Healthy Controls

The comparative analysis between post-GFD (*n* = 88) and CTRLs (*n* = 36) is shown in Table 3. Post-GFD celiac patients exhibited a significantly lower intake of Vitamin D (4.6 ± 9.4 vs. 6.2 ± 12.3 µg/day; *p_adj_* = 0.008), reaching 30.8% of the RI compared to 41.7 in controls (*p_adj_* = 0.009).

A marginal trend towards higher total sugar consumption was observed in the celiac group (*p_adj_* = 0.057). No significant differences were found in macronutrient distribution or Mediterranean Diet adherence (KIDMED score: 6.3 in both groups). Although unadjusted data suggested lower intakes of iron and sodium in celiac patients (*p* < 0.05), these associations disappeared after adjusting for age, sex, and total energy intake (*p_adj_* > 0.05).

### 3.7. Comparative Analysis of Dietary Patterns (NOVA) Between Established GFD and Controls

Daily energy intake from ultra-processed foods (NOVA 4) exceeded 57% in both groups, with no significant differences (p_adj_ = 0.847, Table 4). However, during breakfast, post-GFD celiac patients consumed significantly fewer processed foods (NOVA 3) than CTRLs (3.1 ± 8.4% vs. 11.4 ± 15.9%; p_adj_ = 0.008), while NOVA 4 intake remained similarly high (>74%). Regarding dinner, initial differences in NOVA 1 and NOVA 4 reached only marginal significance or disappeared after adjustment (p_adj_ = 0.078 and p_adj_ = 0.143, respectively).

## 4. Discussion

This study shows the contribution of UPFs to the diet of children with CD, paying particular attention to when these foods are consumed during the day and how intake changes during the first year after starting a GFD. Our main finding is that UPFs were the leading source of daily energy intake not only in children with CD, but also in healthy controls, accounting for more than half of total energy intake in both groups. However, this contribution was not evenly distributed across meals. Breakfast and afternoon snacks were the most affected eating occasions, with UPFs providing approximately three quarters and more than 80% of energy intake, respectively. This pattern indicates that the dietary challenge in pediatric CD goes beyond gluten exclusion and also involves the overall quality of the GFD, the structure of daily meals and the specific moments of the day in which less healthy choices tend to concentrate. These findings may have important implications for dietary counseling in pediatric celiac disease. Rather than focusing exclusively on overall daily UPFs intake, nutritional interventions could target specific eating occasions where UPFs consumption is concentrated. Future studies should identify the individual ultra-processed products that contribute most to energy intake at each meal, particularly during breakfast and afternoon snacks, in order to design more precise and effective dietary interventions aimed at improving the quality of the GFD.

Some studies point to the consumption of (ultra-processed) gluten-free products as the cause of certain micronutrient deficiencies in the celiac diet [24]. However, the results of our study go further, the consumption of UPFs and the consequent deficiency in the intake of important micronutrients, is common to both celiac children and healthy controls.

Regarding the role of the GFD, the results show that, after 12 months following a GFD, there is no substantial modification regarding the nutritional profile or composition of their diet, not even in the Mediterranean diet score, meaning that, apparently, they continue to maintain their dietary habits. Although there is a clear increase in the consumption of simple sugars (34.5 vs. 43.0 g/day, *p* = 0.044) which, although it loses significance when adjusted for confounding variables, remains striking. Celiac children, who already consumed more sugars than healthy ones, have increased their consumption even further with the GFD, which could aggravate the pathophysiology of the disease. This higher consumption of simple sugars with the GFD has also been described for Spanish celiac children by Babio et al. [12] and may be due to the incorporation into their diet of gluten-free products, rich in these carbohydrates [25].

Despite the apparent stability of the nutritional profile, mentioned above, after 12 months on a GFD (Figure 2), celiac subjects do change the foods in their diet, decreasing the use of culinary ingredients (NOVA 2: 9.2 vs. 6.4%; *p_adj_* = 0.029) and processed foods (NOVA 3: 13.3% vs. 9.8%; *p_adj_* = 0.025) in favor of fresh foods and UPFs (54.6% to 58.9%; *p_adj_* = 0.173). This makes sense since, the greater use of fresh foods is logical and characteristic of the GFD, and the higher consumption of UPFs is due to replacing foods that naturally contain gluten with their gluten-free alternatives, which are industrially manufactured products. This higher consumption of UPFs after 12 months of GFD may have contributed to the higher consumption of simple sugars in celiac children (Table 3) compared to healthy children (*p_adj_* = 0.057), coinciding with what was observed by Babio et al. [12].

Our results show that children following a GFD had a lower dietary vitamin D intake than healthy controls (30.8% RI celiacs vs. 41.7% RI controls *p_adj_* = 0.009) (Table 3) and other micronutrients, such as vitamin B5 (12.2% and 15.3% RI), D (30.8% and 41.7%), and E (37.9% and 40.7% RI) and iron (50.7% and 58.4% RI), although these do not reach significance. These deficiencies have been reported by other authors [12].

It is important to highlight the impact of deficient vitamin D concentrations on inflammatory and autoimmune pathologies, where they are associated with higher disease activity. This relationship stems from the fact that the active metabolites of this hormone play a crucial role in reducing inflammation and driving immunomodulation. In its biologically active form, vitamin D binds to the vitamin D receptor (VDR) expressed on both innate and adaptive immune cells, thereby regulating the transcription of immune-response genes. Specifically, vitamin D shifts gene expression away from pro-inflammatory molecules (such as IL-1, IL-8, IL-12, TNFα, interferon-ɣ, and TLR-2 and 4) toward anti-inflammatory cytokines (such as IL-4, IL-5, and IL-10), while promoting regulatory phenotype in macrophages and regulatory T and B cells [26].

Both experimental groups have folate intakes below 50% of the RIs. In the case of celiac subjects, other authors have pointed out that the absence of regulations mandating the folic acid fortification of gluten-free cereals contributes to insufficient folic acid intake among children suffering from CD [27].

Regarding the nutritional profile and composition of the diet at the time of diagnosis, when both experimental groups are following a gluten-containing diet (Table S1), there is no statistically significant difference between them, consistent with the findings of other authors [28]. Both groups (celiac and healthy) present intakes well below the recommended levels for several key nutrients, such as vitamins B5, E, and D, as well as iron (Fe), calcium (Ca), magnesium (Mg), and zinc (Zn) [29], which has already been described by other authors [30]. No differences were found in the Mediterranean diet score either. Notably, the high consumption of simple sugars among the children (around 40 g/day) stands out; this intake is slightly higher in the celiac group and may be associated with dietary patterns potentially relevant to disease development, although causality cannot be inferred.

The anthropometric results (Table 1) show a higher BMI in celiac children (18.5 ± 2.8 vs. 17.1 ± 3.1 kg/m^2^; *p* = 0.025), a finding that becomes even more significant when considering the age difference between the two groups (19.0 ± 3.2 vs. 12.4 ± 2.5 years; *p* = 0.029), given that the WHO growth reference Z-scores are age- and sex-dependent [31]. This places the celiac group on the borderline of overweight (above 19 kg/m^2^ for this age range), whereas the control group remains clearly within the normal weight range (15.5–21.5 kg/m^2^ for this age range). This trend has been previously described in celiac children treated with a GFD [32].

However, some limitations should be acknowledged. First, dietary intake was assessed using family-completed 24 h food records, which may be affected by recall bias, misreporting or underreporting, despite the use of records from three non-consecutive days. Nevertheless, these records were subsequently reviewed by nutritionists prior to analysis. Second, the sample size, particularly for subgroup comparisons and longitudinal analyses, may have limited the statistical power to detect small but clinically relevant differences. Although the analyses were adjusted for sex, our sample size did not allow for robust sex-stratified analyses. Future studies should assess potential sex-related differences in UPF consumption. Third, the control group differed from the celiac group in age and residential distribution; although these variables were considered in the adjusted analyses, residual confounding cannot be completely excluded. Furthermore, the inclusion of patients with different durations of GFD adherence may have introduced some heterogeneity within the post-GFD group. Although GFD adherence was routinely monitored in the clinical cohort using fecal gluten immunogenic peptide testing, individual GIP results were not incorporated into the analyses of the present study. Regarding socioeconomic factors, family income and residential setting were considered, the absence of a more detailed socioeconomic characterization may also have limited our ability to account for socioeconomic influences. Physical activity, sun exposure, and serum 25(OH)D concentrations were not assessed; therefore, the findings reflect dietary vitamin D intake rather than vitamin D status. In addition, although the NOVA classification is widely used to assess the degree of food processing, it does not fully capture differences in nutritional quality among products within the same processing category. Foods classified within the same NOVA category may differ substantially in nutrient composition, food matrix characteristics, and overall healthfulness. Consequently, the degree of processing alone may not fully reflect dietary quality. Therefore, the findings of the present study should be interpreted as reflecting patterns of food processing and meal-specific consumption rather than the overall nutritional adequacy of the diet. Nevertheless, because the primary objective of this study was to characterize the temporal distribution and dietary sources of ultra-processed foods in children with CD following a GFD, the NOVA classification provided an appropriate and widely accepted framework for addressing the research question. Future studies should combine processing-based classifications with nutrient profiling systems and dietary quality indices to provide a more comprehensive assessment of dietary quality in pediatric celiac populations. Finally, this study focused on dietary intake and did not include biological markers of inflammation, microbiota composition or immune activation. Therefore, any mechanistic interpretation regarding the potential inflammatory or immunomodulatory implications of ultra-processed food consumption should be considered indirect and hypothesis-generating.

These findings reinforce the need for long-term nutritional follow-up in children with CD, not only to ensure adherence to the GFD, but also to assess diet quality and make recommendations regarding dietary habits.

## 5. Conclusions

In conclusion, our findings show that the GFD, when not accompanied by structured nutritional guidance, may fail to improve the dietary quality of children with CD and may contribute to the persistence of an already high consumption of UPFs. Although gluten withdrawal remains the cornerstone of treatment, a dietary pattern largely based on ultra-processed gluten-free products may limit the broader nutritional and potentially immunomodulatory benefits expected from a well-balanced GFD. This is particularly relevant given the lower vitamin D intake observed in children with established gluten-free dietary habits and the high contribution of UPFs at breakfast and afternoon snack. Thus, the management of pediatric CD should move beyond the simplistic prescription of “gluten-free diet” as a mere exclusion strategy. Gluten-free dietary treatment should be accompanied by individualized nutritional education and periodic dietary monitoring, aimed not only at preventing gluten exposure but also at improving overall diet quality, reducing ultra-processed food consumption and promoting naturally gluten-free, minimally processed foods. Breakfast and afternoon snacks represent especially relevant windows for intervention.

## Figures and Tables

**Table 1:** Sociodemographic and Anthropometric Characteristics.

Characteristic	Overall *n* = 124 ^1^	CD *n* = 88 ^1^	CTRL *n* = 36 ^1^	*p*-Value ^2^
Sex				>0.9
Female	83 (67%)	59 (67%)	24 (67%)	
Male	41 (33%)	29 (33%)	12 (33%)	
Siblings (nr.)				>0.9
0	13 (11%)	10 (12%)	3 (9.4%)	
1	73 (63%)	52 (62%)	21 (66%)	
2	20 (17%)	14 (17%)	6 (19%)	
3	9 (7.8%)	7 (8.3%)	2 (6.3%)	
4	1 (0.9%)	1 (1.2%)	0 (0%)	
Monthly Income				0.057
€0–999	4 (3.5%)	4 (4.8%)	0 (0%)	
€1000–1499	13 (11%)	10 (12%)	3 (9.4%)	
€1500–2499	47 (41%)	38 (46%)	9 (28%)	
€2500–3499	32 (28%)	19 (23%)	13 (41%)	
€3500–4999	13 (11%)	10 (12%)	3 (9.4%)	
€5000+	6 (5.2%)	2 (2.4%)	4 (13%)	
Residence				0.017
Rural	56 (48%)	34 (40%)	22 (69%)	
Village	37 (32%)	32 (38%)	5 (16%)	
City	24 (21%)	19 (22%)	5 (16%)	
Age (years)	9.2 (3.2)	9.0 (3.2)	12.4 (2.5)	0.029
BMI (kg/m^2^)	17.9 (3.0)	18.5 (2.8)	17.1 (3.1)	0.025
Weight (kg)	37 (13)	40 (12)	35 (14)	0.052
Height (cm)	142 (16)	144 (14)	139 (18)	0.2

^1^ *n* (%); Mean (SD). ^2^ Pearson’s Chi-squared test; Wilcoxon rank sum test. Abbreviations: CD, celiac disease; CTRL, healthy controls; BMI, body mass index; SD, standard deviation.

**Table 2:** Daily and Meal-Specific Energy Distribution by NOVA Food Processing Categories.

Characteristic	CTRL *n* = 36 ^1^	Pre-GFD *n* = 48 ^1^	*p*-unadj ^2^	*p*-adj ^3^
Daily Intake NOVA 1 (%)	23.1 (12.3)	24.5 (12.8)	0.7	0.850
Daily Intake NOVA 2 (%)	7.5 (5.7)	6.9 (5.1)	0.7	0.654
Daily Intake NOVA 3 (%)	12.3 (7.2)	11.3 (7.8)	0.4	0.953
Daily Intake NOVA 4 (%)	57.1 (14.1)	57.3 (16.0)	0.6	0.965
Breakfast Intake NOVA 1 (%)	9.1 (19.3)	16.2 (27.7)	0.2	0.362
Breakfast Intake NOVA 2 (%)	5.4 (8.6)	6.6 (9.2)	0.4	0.128
Breakfast Intake NOVA 3 (%)	11.4 (15.9)	3.2 (7.4)	0.020	0.007
Breakfast Intake NOVA 4 (%)	74.1 (29.9)	74.1 (28.0)	0.8	0.558
Morning Snack Intake NOVA 1 (%)	19.3 (26.6)	11.5 (18.4)	0.074	0.908
Morning Snack Intake NOVA 2 (%)	10.7 (17.8)	7.6 (12.8)	0.7	0.479
Morning Snack Intake NOVA 3 (%)	9.8 (17.4)	13.5 (20.3)	0.5	0.544
Morning Snack Intake NOVA 4 (%)	54.7 (35.3)	65.1 (33.4)	0.2	0.652
Lunch Intake NOVA 1 (%)	42.4 (23.0)	43.4 (24.3)	0.8	0.280
Lunch Intake NOVA 2 (%)	11.9 (12.2)	10.8 (12.1)	0.6	0.906
Lunch Intake NOVA 3 (%)	16.7 (14.7)	20.3 (18.9)	0.5	0.152
Lunch Intake NOVA 4 (%)	29.1 (19.7)	25.6 (19.2)	0.4	0.919
Afternoon Snack NOVA 1 (%)	7.3 (13.3)	10.2 (14.5)	0.5	0.161
Afternoon Snack NOVA 2 (%)	3.2 (8.7)	3.5 (7.8)	>0.9	0.583
Afternoon Snack NOVA 3 (%)	4.9 (9.9)	2.1 (6.2)	0.2	0.195
Afternoon Snack NOVA 4 (%)	81.8 (23.2)	81.9 (21.4)	0.8	0.336
Dinner NOVA 1 (%)	21.7 (20.4)	30.0 (20.8)	0.037	0.101
Dinner NOVA 2 (%)	4.6 (6.7)	5.0 (8.2)	>0.9	0.283
Dinner NOVA 3 (%)	11.5 (13.9)	12.9 (17.2)	0.7	0.611
Dinner NOVA 4 (%)	62.1 (23.4)	52.0 (23.9)	0.060	0.170

^1^ Mean (SD). ^2^ Kruskal–Wallis rank sum test. ^3^ *p*-adjusted calculated via ANCOVA adjusted for age, sex, and total energy intake (kcal/day). Abbreviations: CTRL, healthy controls; GFD, gluten-free diet; SD, standard deviation.

**Table 3:** Comparative analysis of nutritional intake between celiac patients with established gluten-free dietary patterns (*n* = 88) and healthy controls (*n* = 36).

Characteristic	CTRL *n* = 36 ^1^	Post-GFD *n* = 88 ^1^	*p*-unadj ^2^	*p*-adj ^3^
Energy Intake (kcal/day)	1806.7 (351.2)	1839.4 (408.1)	0.9	0.223
Protein (g)	76.6 (17.9)	77.4 (23.8)	>0.9	0.284
Total Fat (g)	599.2 (196.3)	673.9 (220.7)	0.2	0.391
Saturated Fat (g)	24.4 (7.4)	24.9 (9.1)	>0.9	0.539
Cholesterol (mg)	230.8 (111.7)	217.2 (116.4)	0.5	0.898
Carbohydrates (g)	817.5 (213.0)	791.6 (200.3)	0.5	0.192
Total Sugar (g)	39.8 (22.4)	40.6 (19.5)	0.8	0.057
Fiber (g)	11.9 (4.3)	13.2 (5.6)	0.2	0.243
Fiber (% RI) *	67.9 (23.1)	76.5 (34.3)	0.3	0.159
Vitamin A (μg retinol eq)	332.1 (281.2)	329.3 (291.4)	0.7	0.418
Vitamin A (% RI) *	75.4 (70.7)	72.1 (70.0)	0.7	0.337
Vitamin B6 (mg)	1.0 (0.4)	1.1 (0.5)	0.6	0.093
Vitamin B6 (% RI) *	94.9 (50.4)	100.2 (72.3)	0.8	0.163
Vitamin B12 (μg)	2.9 (2.0)	3.0 (2.9)	0.5	0.541
Vitamin B12 (% RI) *	111.5 (82.7)	108.7 (98.5)	0.4	0.380
Vitamin C (mg)	46.6 (35.2)	54.9 (39.5)	0.2	0.233
Vitamin C (% RI) *	94.2 (81.5)	111.2 (104.5)	0.4	0.269
Vitamin D (μg)	6.2 (12.3)	4.6 (9.4)	0.6	0.008
Vitamin D (% RI) *	41.7 (81.9)	30.8 (62.6)	0.6	0.009
Vitamin E (mg tocoferol)	4.2 (2.9)	4.0 (2.4)	0.9	0.820
Vitamin E (% RI) *	40.7 (29.5)	37.9 (22.6)	>0.9	0.841
Iron (mg)	6.3 (3.5)	5.4 (3.5)	0.029	0.787
Iron (% RI) *	58.4 (33.0)	50.7 (37.6)	0.029	0.877
Calcium (mg)	428.6 (210.0)	373.7 (245.7)	0.2	0.397
Calcium (% RI) *	46.6 (40.5)	34.8 (24.7)	0.087	0.440
Potassium (mg)	1481.8 (574.0)	1487.0 (669.0)	0.8	0.093
Potassium (% RI) *	78.3 (43.1)	76.5 (52.1)	0.4	0.099
Sodium (mg)	1419.4 (772.0)	1193.0 (810.5)	0.036	0.108
Folate (μg)	97.3 (39.8)	100.1 (49.2)	>0.9	0.410
Folate (% RI) *	45.0 (17.8)	47.0 (30.5)	0.7	0.235
Magnesium (mg)	123.2 (54.9)	120.6 (61.3)	0.6	0.188
Magnesium (% RI) *	52.2 (28.1)	49.4 (30.1)	0.3	0.302
Zinc (mg)	4.2 (1.5)	4.0 (2.0)	0.2	0.580
Zinc (% RI) *	52.1 (24.5)	48.2 (29.6)	0.2	0.298
Adherence to Mediterranean Diet	6.3 (2.1)	6.3 (2.1)	0.6	0.082

^1^ Mean (SD). ^2^ Kruskal–Wallis rank sum test. ^3^ *p*-adjusted calculated via ANCOVA adjusted for age, sex, and total energy intake (kcal/day). * RI: Reference Intake based on EFSA guidelines. Abbreviations: CTRL, healthy controls; GFD, gluten-free diet; RI, reference intake; SD, standard deviation.

**Table 4:** Distribution of daily and occasion-specific energy intake according to NOVA groups: Comparison between patients with established gluten-free diet (*n* = 88) and healthy controls (*n* = 36).

Characteristic	CTRLs *n* = 36 ^1^	Post-GFD *n* = 88 ^1^	*p*-unadj ^2^	*p*-adj ^3^
Daily Intake NOVA 1 (%)	23.1 (12.3)	24.5 (12.1)	0.6	0.832
Daily Intake NOVA 2 (%)	7.5 (5.7)	6.7 (5.2)	0.4	0.722
Daily Intake NOVA 3 (%)	12.3 (7.2)	10.6 (7.8)	0.094	0.791
Daily Intake NOVA 4 (%)	57.1 (14.1)	58.3 (14.7)	0.4	0.847
Breakfast Intake NOVA 1 (%)	9.1 (19.3)	13.4 (25.3)	0.5	0.454
Breakfast Intake NOVA 2 (%)	5.4 (8.6)	5.8 (10.0)	>0.9	0.197
Breakfast Intake NOVA 3 (%)	11.4 (15.9)	3.1 (8.4)	0.002	0.008
Breakfast Intake NOVA 4 (%)	74.1 (29.9)	76.5 (30.3)	0.8	0.711
Morning Snack Intake NOVA 1 (%)	19.3 (26.6)	10.6 (17.9)	0.027	0.998
Morning Snack Intake NOVA 2 (%)	10.7 (17.8)	7.2 (12.0)	0.5	0.524
Morning Snack Intake NOVA 3 (%)	9.8 (17.4)	9.2 (17.4)	0.6	0.868
Morning Snack Intake NOVA 4 (%)	54.7 (35.3)	64.0 (34.8)	0.2	0.701
Lunch Intake NOVA 1 (%)	42.4 (23.0)	41.0 (23.0)	0.9	0.165
Lunch Intake NOVA 2 (%)	11.9 (12.2)	10.7 (12.3)	0.5	0.885
Lunch Intake NOVA 3 (%)	16.7 (14.7)	18.0 (17.6)	0.8	0.202
Lunch Intake NOVA 4 (%)	29.1 (19.7)	30.4 (20.3)	0.8	0.535
Afternoon Snack NOVA 1 (%)	7.3 (13.3)	11.8 (19.7)	0.6	0.226
Afternoon Snack NOVA 2 (%)	3.2 (8.7)	3.3 (7.7)	0.9	0.604
Afternoon Snack NOVA 3 (%)	4.9 (9.9)	2.5 (6.8)	0.13	0.261
Afternoon Snack NOVA 4 (%)	81.8 (23.2)	79.0 (26.8)	0.7	0.310
Dinner NOVA 1 (%)	21.7 (20.4)	31.8 (21.4)	0.007	0.078
Dinner NOVA 2 (%)	4.6 (6.7)	4.2 (6.7)	0.8	0.305
Dinner NOVA 3 (%)	11.5 (13.9)	12.6 (15.0)	0.6	0.526
Dinner NOVA 4 (%)	62.1 (23.4)	51.4 (23.6)	0.020	0.143

^1^ Mean (SD). ^2^ Wilcoxon rank sum test. ^3^ *p*-adjusted calculated via ANCOVA adjusted for age, sex, and total energy intake (kcal/day). Abbreviations: CTRL, healthy controls; GFD, gluten-free diet; SD, standard deviation.

## Data Availability

The data presented in this study are available on request from the corresponding author due to due to data protection restrictions and ethical constraints.

## Supplementary Materials

The following supporting information can be downloaded at: https://www.mdpi.com/article/10.3390/nu18132173/s1, Table S1: Nutritional Intake and Mediterranean Diet Adherence.
