# Supplementary material for: Patterns of Ultra-Processed Food Consumption in a Gluten-Free Diet: A Target for Nutritional Intervention
Source: Nutrients. 2026 Jul 4;18(13):2173. doi: 10.3390/nu18132173 (PMC13363960; doi:10.3390/nu18132173)
Supplement: Supplementary file 1 [file nutrients-18-02173-s001.zip › nutrients-4379879-supplementary.pdf]

Table S1. Nutritional Intake and Mediterranean Diet Adherence

Characteristic	CTRL N = 36[1]	pre-GFD N = 44[1]	p-unadj[2]	p-adj*[3]
Energy Intake (kcal/day)	1,806.7 (351.2)	1,850.7 (460.0)	0.7	0.256
Protein (g)	76.6 (17.9)	77.1 (26.1)	0.8	0.367
Total Fat (g)	599.2 (196.3)	681.5 (206.8)	0.2	0.216
Saturated Fat (g)	24.4 (7.4)	25.3 (9.4)	0.7	0.655
Cholesterol (mg)	230.8 (111.7)	209.8 (113.8)	0.4	0.785
Carbohydrates (g)	817.5 (213.0)	809.6 (216.7)	0.7	0.121
Total Sugar (g)	39.8 (22.4)	40.1 (17.2)	0.8	0.052
Fiber (g)	11.9 (4.3)	14.0 (6.4)	0.15	0.173
Fiber (% RI)[4]	67.9 (23.1)	80.6 (38.7)	0.2	0.137
Vitamin A (ug retinol eq)	332.1 (281.2)	354.5 (299.6)	0.7	0.318
Vitamin A (% RI)[4]	75.4 (70.7)	75.5 (70.5)	0.9	0.285
Vitamin B6 (mg)	1.0 (0.4)	1.0 (0.5)	0.8	0.168
Vitamin B6 (% RI)[4]	94.9 (50.4)	88.6 (49.0)	0.5	0.140
Vitamin B12 (ug)	2.9 (2.0)	3.0 (3.2)	0.4	0.597
Vitamin B12 (% RI)[4]	111.5 (82.7)	99.7 (98.5)	0.2	0.483
Vitamin C (mg)	46.6 (35.2)	59.6 (45.5)	0.2	0.221
Vitamin C (% RI)[4]	94.2 (81.5)	109.9 (92.9)	0.3	0.258
Vitamin D (ug)	6.2 (12.3)	5.5 (12.2)	0.9	0.070
Vitamin D (% RI)[4]	41.7 (81.9)	36.4 (81.1)	0.8	0.070
Vitamin E (mg tocoferol)	4.2 (2.9)	4.2 (2.6)	0.6	0.989
Vitamin E (% RI)[4]	40.7 (29.5)	37.9 (21.4)	0.9	0.845
Iron (mg)	6.3 (3.5)	5.4 (4.0)	0.034	0.804
Iron (% RI)[4]	58.4 (33.0)	48.8 (38.0)	0.025	0.972
Calcium (mg)	428.6 (210.0)	377.0 (222.1)	0.4	0.323
Calcium (% RI)[4]	46.6 (40.5)	33.0 (19.7)	0.11	0.454
Potassium (mg)	1,481.8 (574.0)	1,485.1 (676.5)	0.8	0.117
Potassium (% RI)[4]	78.3 (43.1)	69.8 (35.8)	0.4	0.070
Sodium (mg)	1,419.4 (772.0)	1,174.7 (841.6)	0.046	0.132
Folate (ug)	97.3 (39.8)	105.5 (54.8)	0.6	0.330
Folate (% RI)[4]	45.0 (17.8)	46.1 (24.6)	>0.9	0.174
Magnesium (mg)	123.2 (54.9)	120.9 (64.0)	0.6	0.226
Magnesium (% RI)[4]	52.2 (28.1)	47.1 (25.4)	0.3	0.318
Zinc (mg)	4.2 (1.5)	3.8 (1.9)	0.2	0.681
Zinc (% RI)[4]	52.1 (24.5)	44.1 (23.4)	0.14	0.375
Adherence to Mediterranean Diet (score)	6.3 (2.1)	6.4 (2.2)	0.8	0.078

[1]Mean (SD)
[2]Kruskal-Wallis rank sum test
[3]P-adjusted calculated via ANCOVA. Energy variables adjusted for age and sex. Nutrients and percentages additionally adjusted for total energy intake (kcal/day).
[4]RI: Reference Intake based on EFSA guidelines
